# Extracellular traps, an ancient defense mechanism described in hemocytes of the tick *Rhipicephalus microplus*

**DOI:** 10.1186/s13071-025-07165-4

**Published:** 2025-12-12

**Authors:** Hugo Aguilar-Díaz, Rosa Estela Quiroz-Castañeda, Karina Salazar-Morales, César Díaz-Godínez, Raquel Cossío-Bayúgar, Julio César Carrero, Estefhan Miranda-Miranda, Salvador Hernández-Martínez

**Affiliations:** 1 Centro Nacional de Investigación Disciplinaria en Salud Animal e Inocuidad, Instituto Nacional de Investigaciones Forestales, Agrícolas y Pecuarias (CENID-SAI, INIFAP), Jiutepec, México; 2 Centro de Investigaciones Sobre Enfermedades Infecciosas, Instituto Nacional de Salud Pública, Cuernavaca, MOR México; 3 Instituto de Investigaciones Biomédicas, UNAM, Mexico City, México

**Keywords:** *Rhipicephalus microplus*, Hemocytes, Extracellular traps, ETosis, Immune response, PMA, Zymosan A, LPS, A23817

## Abstract

**Background:**

NETosis is a conserved process that has been maintained throughout evolution in various species. However, in the cattle tick *Rhipicephalus microplus*, a process similar to NETosis, known as ETosis, has not been previously described.

**Methods:**

In this work, we demonstrate, using fluorometry and confocal and electron microscopy, the chromatin release and the extracellular trap (ET) formation in tick hemocytes in response to various treatments.

**Results:**

The treatments analysis showed greater chromatin release in zymosan A-, *Escherichia coli*-, and LPS-treated hemocytes. This was consistent with the expression of the peroxinectin gene (*pxn*), the myeloperoxidase (*mpo*) analog gene in vertebrates, which participates in NETosis activation. Furthermore, DNA fibers were observed in tick hemocytes under all treatments, and transmission electron microscopy (TEM) showed that hemocytes treated with zymosan A have a clear nuclear envelope disruption, with a unidirectional release of chromatin.

**Conclusions:**

This work investigates the existence of ETosis in tick hemocytes, representing a significant step toward understanding the tick’s immune response. In addition to this contribution, new areas of research are emerging to understand the molecular mechanisms that govern this process, which we are currently exploring.

**Graphical Abstract:**

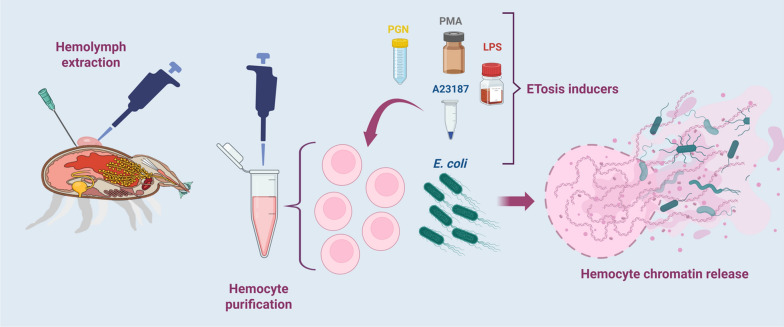

## Background

Neutrophils also known as polymorphonuclear granulocytes (PMNs) are the first line of defense in the innate immune response against invading pathogens, and display mechanisms to combat microorganisms, including, phagocytosis, degranulation, reactive oxygen species (ROS) production, chemotaxis, and neutrophil extracellular traps release neutrophil extracellular traps (NETs), a process known as NETosis [1]. NETs are web-like structures formed by DNA, histones (H2A, H2B, H3, and H4), and granules with protease activity that trap and kill pathogens, thereby preventing their dissemination [2]. The specific composition of neutrophil granules comprises bactericidal proteins, such as histones, proteases (neutrophil elastase [NE], myeloperoxidase [MPO], cathepsin C, and cathepsin G), and other proteases, as well as cytoplasmic proteins calprotectin and lactoferrin [1]. Some antimicrobial proteins, such as azurocidin, cathelicidin, lysozyme C, and BPIF2, have also been reported in NETosis [3]. NETosis comprises two types: suicidal and vital. In suicidal NETosis, the nuclear and cell membrane disruption and lysogenic cell death are the main characteristics. In contrast, vital NETosis involves vesicle formation to transport traps without any nuclear and cell membrane damage [4]. The main differences between both processes are the type of stimuli, the trap release time, and the reactive oxygen species (ROS) production (NOX-dependent/independent or mitochondrial) [4]. Neutrophils are not the only cells performing NETosis; macrophages, eosinophils, basophils, and mast cells also release extracellular traps (ETs) through a process called ETosis [5, 6]. Similarly to NETs, ETs are triggered by pathogen-associated molecular patterns (PAMPs) of bacteria, fungi, parasites, and viruses [7, 8]. Interestingly, ETosis occurs in several arthropod species, where the released chromatin and histones by hemocytes may represent an ancient defense mechanism against pathogens [9]. The first evidence of ETs formation in invertebrates was reported in hemocytes of the moth *Galleria mellonella*, which released ETs in response to *Escherichia coli* infection, and in shrimp *Litopenaeus vannamei* hemocytes, which formed ETs in response to *E. coli*, lipopolysaccharide (LPS) and chemical inducer phorbol myristate acetate (PMA) [7, 10]. In addition, there are reports of ET formation in the NOX-dependent pathway in the crab *Carcinus maenas* and the bivalve mollusk *Crassostrea gigas,* induced by bacterial pathogens and PMA, and zymosan A, respectively [9, 11]. Also, it was reported that ETs can be activated by NOX-dependent and -independent mechanisms in the mollusk *Mytilus galloprovincialis,* and induced by different stimuli, such as zymosan A, calcium ionophore A23187, and ultraviolet (UV) [12]. This evidence suggests that ETosis is an evolutionarily conserved mechanism that is not exclusive to vertebrates, as it has been reported in several invertebrates species [12–14]*.* Nevertheless, the molecules participating in invertebrate ETosis and the mechanisms are yet to be clarified.

Specifically, in ticks, the innate immune response comprises cellular responses, such as phagocytosis and encapsulation, as well as humoral responses, including defensins, antimicrobial peptides, proteases, and the cascades that regulate the coagulation of hemolymph [15, 16]. Nevertheless, the ET formation has not been reported in the cattle tick *Rhipicephalus microplus* [17]. Thus, the presence of specific proteins involved in ETosis in *R. microplus*, including MPO, neutrophil elastase (NE), defensins, and histones, is significant in elucidating and proposing how this mechanism functions in the triad host–vector–pathogen. In this regard, it has been proposed that proteins involved in chromatin decondensation, such as HSP27 and peroxinectin (PXN), would act similarly to myeloperoxidase in neutrophils. At the same time, cathepsins D and L might perform a similar function to the antimicrobial peptide cathepsin C and/or G of vertebrates [18–22].

In this work, we demonstrate the chromatin release in *R. microplus* hemocytes triggered by chemical and biological inducers, as well as *E. coli*. In addition, we assessed the relative expression of the peroxinectin gene (*pxn*) in hemocytes, and chromatin release was visualized by laser scanning confocal microscopy (LCSM) and transmission electron microscopy (TEM). Our results strongly support the ETosis mechanism existence in *R. microplus* hemocytes, which could represent an alternative to identify new targets for developing strategies to control ticks and tick-borne diseases. Finally, this contribution reinforces the occurrence of ETosis in invertebrate species.

## Methods

### Biological material

Semi-engorged female *R. microplus* ticks were collected from naturally infested 6-month Angus male bovines from Las Margaritas ranch in Tapalpa, Jalisco, Mexico. The ticks were rinsed once with tridistilled water, washed three times by immersion in 10% benzalkonium chloride (Química Meyer, CDMX, Mexico), and then rinsed. The last wash was carried out with a mixture of distilled water and antibiotic/antimycotic (100X, 10,000 units/mL penicillin units, 10,000 μg/mL of streptomycin, and 25 μg/mL of amphotericin B, Gibco™, Waltham, MA, USA), at a ratio of 1:100 and rinsed with sterile tridistilled water. Finally, the ticks were dried on absorbent sterile paper and stored at 4 °C overnight to increase the extraction yield of hemocyte cells [23].

### Hemocyte purification

The hemolymph extraction was performed according to the protocol reported in [23]. For the hemocyte purification, the hemolymph from a pool of 20 ticks was extracted by hipocuticular puncture. A volume of 1.5 mL hemolymph was obtained and resuspended in 100 μL Alsever’s modified anticoagulant citrate buffer at 4 °C (anhydrous α-d-glucose 20.8 g/L, trisodium citrate 8 g/L, ethylenediaminetetraacetic acid (EDTA) dihydrate 3.36 g/L, NaCl 22.3 g/L, pH 7.4, Sigma Aldrich, St Louis MO, USA) and then centrifuged at 2000*g* for 1 min. The plasma was discarded, and the cell pellet was resuspended in MEM/L-15 medium (11935046 and 21083027, Fisher Scientific, Waltham, MA, USA) supplemented with 5% of heat-inactivated fetal bovine serum (FBS; A5670701 Thermo Fisher, Waltham, MA, USA).

### Chemical, biological, and bacterial inducers

Extracellular trap inducers used were phorbol 12-myristate 13-acetate (PMA; P1584 Sigma Aldrich, St Louis MO, USA) and calcium ionophore (A23187, C7522 Sigma Aldrich, St Louis MO, USA), considered as chemical inducers; zymosan A from cell walls of *Saccharomyces cerevisiae* (Z4250, Sigma Aldrich, St Louis MO, USA); peptidoglycan from *Staphylococcus aureus* (PGN, 77140 Sigma Aldrich, St Louis MO, USA); and lipopolysaccharide from *Escherichia coli* serotype O128:B12 (LPS, L2755 Sigma Aldrich, St Louis MO, USA) (biological inducers) as well as *E. coli* cells (bacterial inducer). PMA was used at 0.005, 0.010, 0.020, and 0.050 µM; A23187 was used at 1, 5, 10, and 20 µM; LPS and PGN were used at 1, 5, 10, and 20 µg/mL; and zymosan A was used at 0.00125, 0.0025, 0.005, and 0.01 µM. Finally, *E. coli* was used at concentrations of 1 × 10^5^, 2.5 × 10^5^, 5 × 10^5^, and 7.5 × 10^5^ cells. A 10% Triton X-100 solution (Thermo Scientific, Waltham, MA, USA) was used as a positive control for DNA released by cellular lysis, and untreated hemocytes (without inducer treatment) served as a negative control. All treatments were performed at 0, 5, 10, 15, 30, 60, 120, and 180 min.

### Quantification of chromatin release

The viability of tick hemocytes was assessed by excluding dying cells with trypan blue (Sigma-Aldrich, St. Louis, MO, USA) and counted using a CYTOsmart Corning Cell Counter, 1 × 10^5^. Hemocytes were placed in a 96-well polystyrene plate with 1 μM Sytox-Green (Thermo Fisher, Waltham, MA, USA) and adjusted to 100 μL of MEM/L-15 medium. Untreated hemocytes were used as a negative control. After adding the inducers, the cells were allowed to sediment on the bottom of the plate for 20 min at room temperature, followed by reads at 0, 15, 30, 60, 120, and 180 min at 30 °C using spectrofluorometer Biotek Synergy HTX (Agilent, Santa Clara, CA, USA), with 485-nm excitation and 528-nm emission filters.

### Laser scanning confocal microscopy (LSCM)

Chromatin fluorescent staining was performed by hemocytes stimulated with different inducers after 30 min of treatment (the optimal time for chromatin release). The cells were placed in a LabTek Chamber Slide (Thermo Fisher, Waltham, MA, USA) with 200 µL MEM/L-15 medium and incubated at 30 °C in a 5% CO_2_ atmosphere. Before the incubation treatments, the cells were centrifuged for 30 min at room temperature in a humid chamber. The concentrations used were 20 µM A23187, 0.050 µM PMA, 20 µg/mL LPS, 20 µg/mL PGN, 0.01 µM zymosan A, 7.5 × 10^5^ *E. coli* cells, and 10% Triton X-100. All treatments were incubated at 30 °C for 30 min. After each incubation, cells were fixed with 4% paraformaldehyde (Sigma Aldrich, St Louis, MO, USA), PBS (137 mM NaCl, 2.7 mM KCl, 10 mM Na_2_HPO_4_, 2 mM KH_2_PO_4_, pH 7.4) for 30 min. After the specified time had elapsed, the wells were washed three times with PBS (pH 7.4), and the chamber cover was removed. The preparations were mounted with DAPI Fluoromount-G^TM^ (Thermo Fisher, Waltham, MA, USA). They were observed with the NIS-Elements C Imaging software of a confocal system (Nikon C2, Japan) coupled to a Nikon E-600 microscope (Melville, NY, USA) for analysis and documentation.

### Transmission electron microscopy (TEM) of tick hemocytes

The ultrastructure of tick hemocytes was assessed under both unstimulated and stimulated conditions. After incubation with 0.01 mM zymosan and 10% Triton X-100 for 15 min, the hemocytes were fixed by incubating in Karnovsky’s fixative (16% paraformaldehyde, 50% glutaraldehyde, and 0.2 M sodium phosphate buffer) for 72 h at 4 °C. Subsequently, cells were washed with 0.1 M sodium cacodylate buffer (Sigma Aldrich, St. Louis, MO, USA), followed by 4 μL of 0.1% osmium tetroxide in Zelterqust buffer. The samples’ dehydration was performed using ethanol at different concentrations (70%, 80%, 90%, 95%, and 100%, twice for 10 min each), and they were then incubated in acetonitrile (Sigma Aldrich, St. Louis, MO, USA) for 20 min. Subsequently, the samples were embedded in epoxy resin (EPON) (Merck Millipore, St. Louis, MO, USA) and cut into ultrathin sections using a Minot-type microtome. The sections were contrasted with 5% uranyl acetate (Fisher Scientific, Waltham, MA, USA) in distilled water and 0.25% lead citrate (Fisher Scientific) in 0.1 N NaOH (Sigma-Aldrich, St. Louis, MO, USA). The samples were observed in a JEOL 1010 transmission electron microscope at the Faculty of Sciences (UNAM).

### RNA extraction from tick hemocytes

Total RNA from untreated and tick hemocytes (1 × 10^5^) treated with 20 µM A23187, 0.050 µM PMA, 20 µg/mL PGN, 20 µg/mL LPS, 0.01 µM zymosan A, 7.5 × 10^5^ *E. coli* cells, and 10% Triton X-100 was obtained by conventional Trizol™ reagent protocol (Invitrogen, Carlsbad, CA, USA), following the manufacturer’s instructions. The RNA was resuspended in nuclease-free tridistilled water and stored at −70 °C until use. The total RNA quantification was performed using a Nabi UV/Vis Nanospectrophotometer (Microdigital, Seoul, South Korea), and the integrity was evaluated by electrophoresis on a 2% agarose gel for 30 min at 120 V.

### Semi-quantitative reverse transcription polymerase chain reaction (sqRT–PCR) of peroxinectin (pxn)

The *pxn* relative expression levels were determined by sqRT–PCR, using 200 ng total RNA after 30 min of each treatment. The reverse transcription (RT) reaction was performed using the RevertAid First Strand cDNA Synthesis Kit (Thermo Scientific, Waltham, MA, USA) according to the manufacturer’s instructions in a Thermocycler (Techne TC-412, Keison Products, Grants Pass, OR, USA). The complementary DNA (cDNA) synthesized was quantified in a Nabi UV/Vis Nanospectrophotometer (Microdigital, Seoul, South Korea) and used as template in a PCR reaction to amplify a partial sequence of 900 bp of the *R. microplus pxn* gene under the following conditions: an initial denaturation for 5 min at 94 °C, followed by 30 cycles of denaturation for 30 s at 94 °C, annealing for 1 min at 58 °C, and extension for 1 min at 72 °C. A final extension was performed for 5 min at 72 °C. The oligonucleotide sequences used were pxnFwd: 5′-ATGCAGATCCTTCTCTTGAG-3′ and pxnRev: 5′-GTTCGGAAAATCAGCGTCGG-3′, with a Tm of 52 °C and 56 °C, respectively. In addition, a partial sequence of 690 bp of the *R. microplus* phospholipid-hydroperoxide glutathione peroxidase (*phgpx*) housekeeping gene was amplified by a PCR reaction set as follows: an initial denaturation for 5 min at 94 °C, followed by 30 cycles of denaturation for 1 min at 94 °C, annealing for 30 s at 60 °C, and extension for 30 s at 72 °C. A final extension was performed for 5 min at 72 °C. The primers used were: phgpxFwd: 5′-GCTAGACTGCACAAGCAATACGGG-3′, and phgpxRev: 5′-GTTTGCAGACACCTCAGCGTGCC-3′, with a Tm of 60 °C. The PCR reactions for both amplifications were prepared as follows: 12.5 µL GoTaq^®^ Green Mastermix 2X (Promega, Madison, WI, USA), 0.5 µL (10 pmol/µL) of each oligonucleotide, 1 µL (500 ng) cDNA template, and adjusted with nuclease-free water to a final volume of 25 µL. All PCR products were visualized on a 1.5% agarose gel, and densitometric analyses were carried out using the calculated area and pixel value statistics of user-defined selections in ImageJ 1.8.0 software, accessed on 5 May 2025 [24].

### Statistical analysis

The data were normalized with the negative controls. The statistical significance of the data was determined using two-way analysis of variance (ANOVA) with a post hoc Tukey test and a Dunnett’s multiple comparisons test performed with the GraphPad Prism 8 program (GraphPad Software, Boston, MA, USA). *P* values < 0.0001 were considered statistically significant. All measurements were conducted in triplicate (*n* = 3) with three internal repetitions.

## Results

### Tick hemocytes release chromatin

**Fig. 1:**
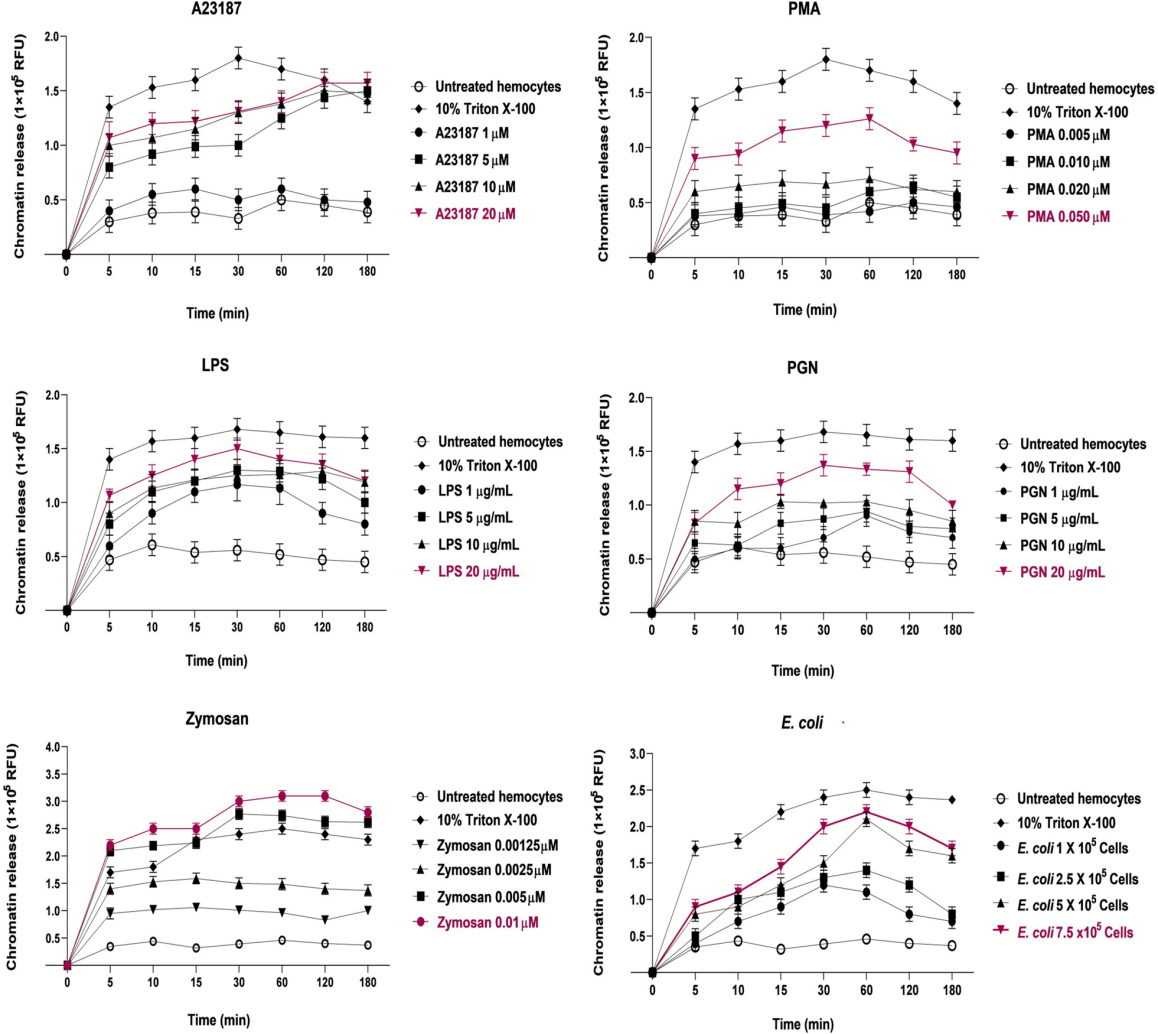
Kinetics of hemocyte chromatin release treated with different ETosis inducers. Chemical inducers: **A** A23817 and **B** PMA. Biological inducers: **C** LPS; **D** PGN; and **E** zymosan A. **F** Bacterial inducer: *E. coli* cells. The kinetics was performed from 0 to 180 min. Untreated hemocytes were used as a negative control, and 10% Triton X-100 was used as a positive control to release DNA through cellular lysis. The magenta line indicates the optimal concentration of the inducer. RFU, relative fluorescence units. Two-way ANOVA with Tukey’s multiple comparisons test post hoc was performed. The significant difference (^****^P < 0.0001) refers to the comparison between hemocytes treated with the best concentration inducing chromatin release and untreated hemocytes (control group)

To determine if tick hemocytes release chromatin similar to ETosis, we treated hemocytes with different inducers. Our results revealed that the higher concentration used for chemical, biological, and *E. coli* cells stimulated the major chromatin release in hemocytes in a time- and concentration-dependent way. Specifically, the chemical inducers A23817 (20 µM) and PMA (0.050 µM) exhibited maximum chromatin release at 120 and 60 min, respectively (Fig. 1A, B). However, the biological inducers LPS and PGN also stimulated hemocyte chromatin release at 20 µg/mL after 30 min (Fig. 1C, D). Notably, zymosan A was the best inducer, as it showed that 0.01 µM triggered a peak of chromatin release at 120 min, even higher than 10% Triton X-100 (Fig. 1E). Lastly, the induction with 7.5 × 10^5^ *E. coli* cells resulted in a peak of chromatin release at 60 min (Fig. 1F).

**Fig. 2:**
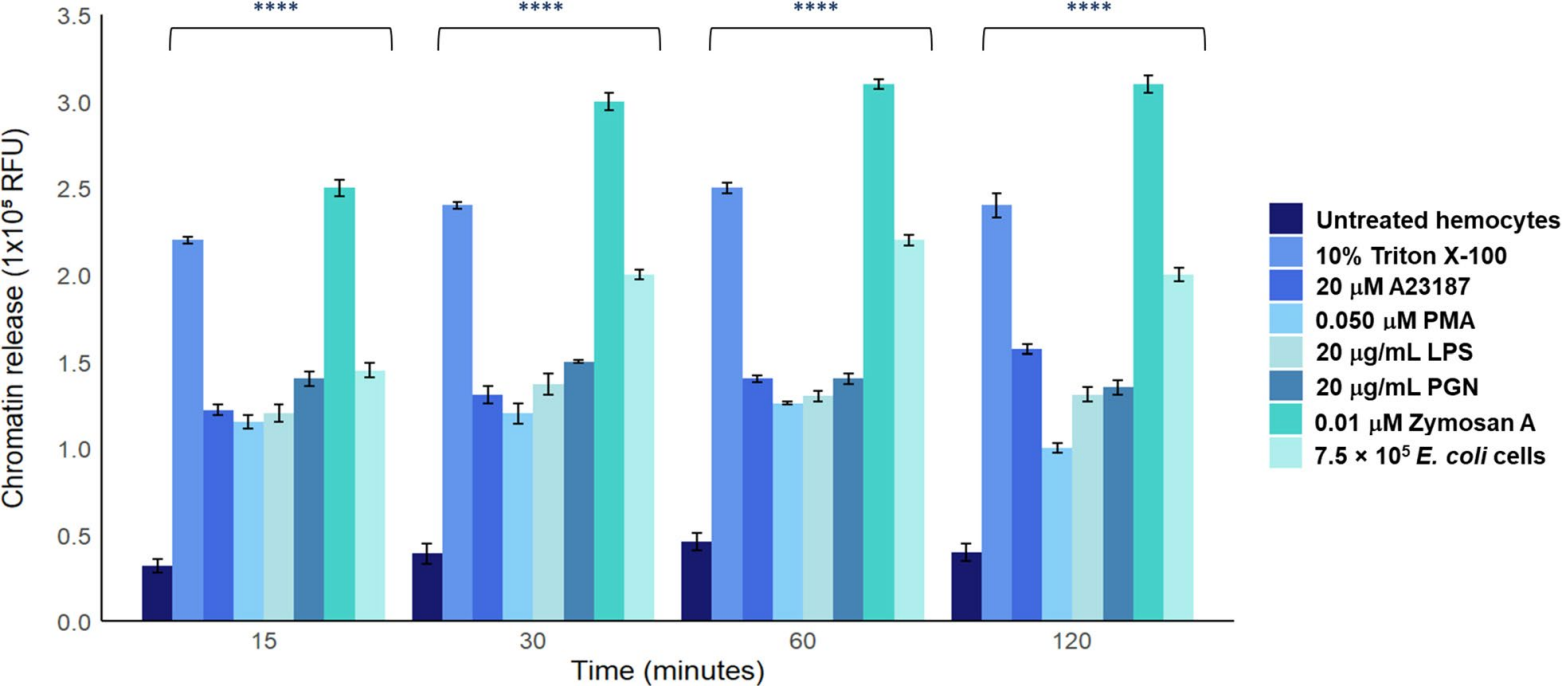
Comparison of the best chromatin release concentrations in the optimal time range (15–120 min) for each inducer. The zymosan A, LPS, and *E. coli* cells showed significant differences compared with untreated hemocytes. Two-way ANOVA with Dunnett’s multiple comparisons test post hoc was performed. ^****^P < 0.0001. RFU, relative fluorescence units

In addition, we compared the optimal concentrations for each inducer during the optimal period of chromatin release (that is, from 15 to 120 min). Tick hemocytes treated with zymosan A (0.01 µM), followed by *E. coli* (7.5 × 10^5^ cells) and LPS (20 µg/mL) exhibited the best chromatin release compared with negative control (untreated hemocytes) (Fig. 2).

### Peroxinectin (pxn) gene is expressed in tick hemocytes

**Fig. 3:**
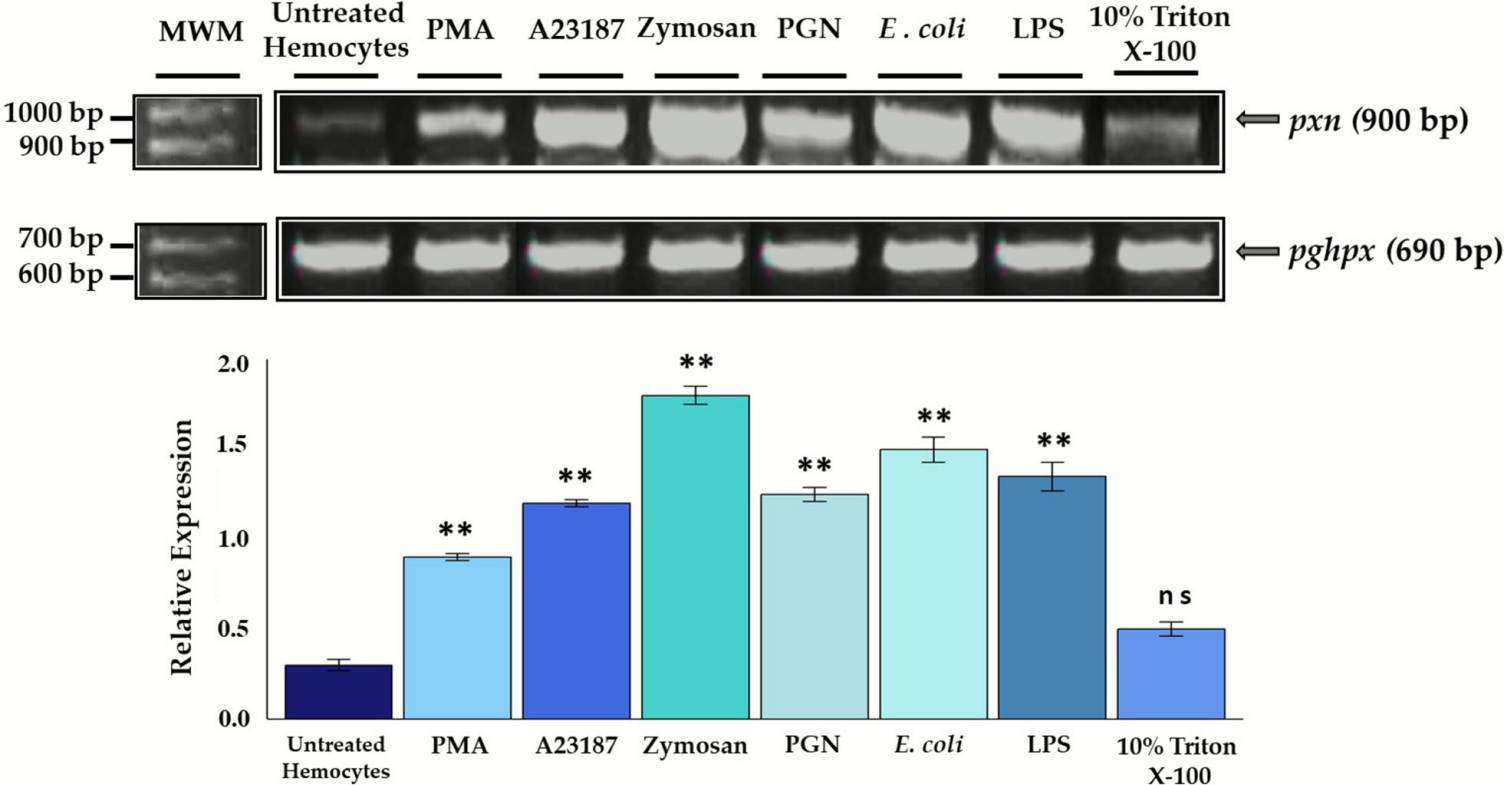
Determination of *pxn* relative expression by sqRT–PCR in tick hemocytes at early times of chromatin release. All treatments showed significant differences compared with untreated hemocytes, except 10% Triton X-100. The *R. microplus pghpx* was used as an endogenous control for normalizing the *pxn* gene’s relative expression. One-way ANOVA with Tukey’s multiple comparison post hoc test compared with control. ^**^P < 0.001; ns, not significant

Peroxidases play essential roles as mediators of innate immune responses, and in arthropods, PXN acts as an MPO homolog, as mentioned elsewhere. Therefore, we decided to assess the expression of the *pxn* gene by sqRT–PCR at early times (10 min), when the hemocytes are still viable and preparing the molecular machinery to release the chromatin traps. We found that all inducers showed *pxn* overexpression compared with untreated hemocytes. Interestingly, and in line with our previous results, zymosan A*, E. coli* cells, and LPS were the most effective inducers of *pxn* expression (Fig. 3).

### Laser scanning confocal microscopy (LSCM) analysis of tick hemocytes treated with A23187, PMA, LPS, PGN, zymosan A, and *E. coli* cells

**Fig. 4:**
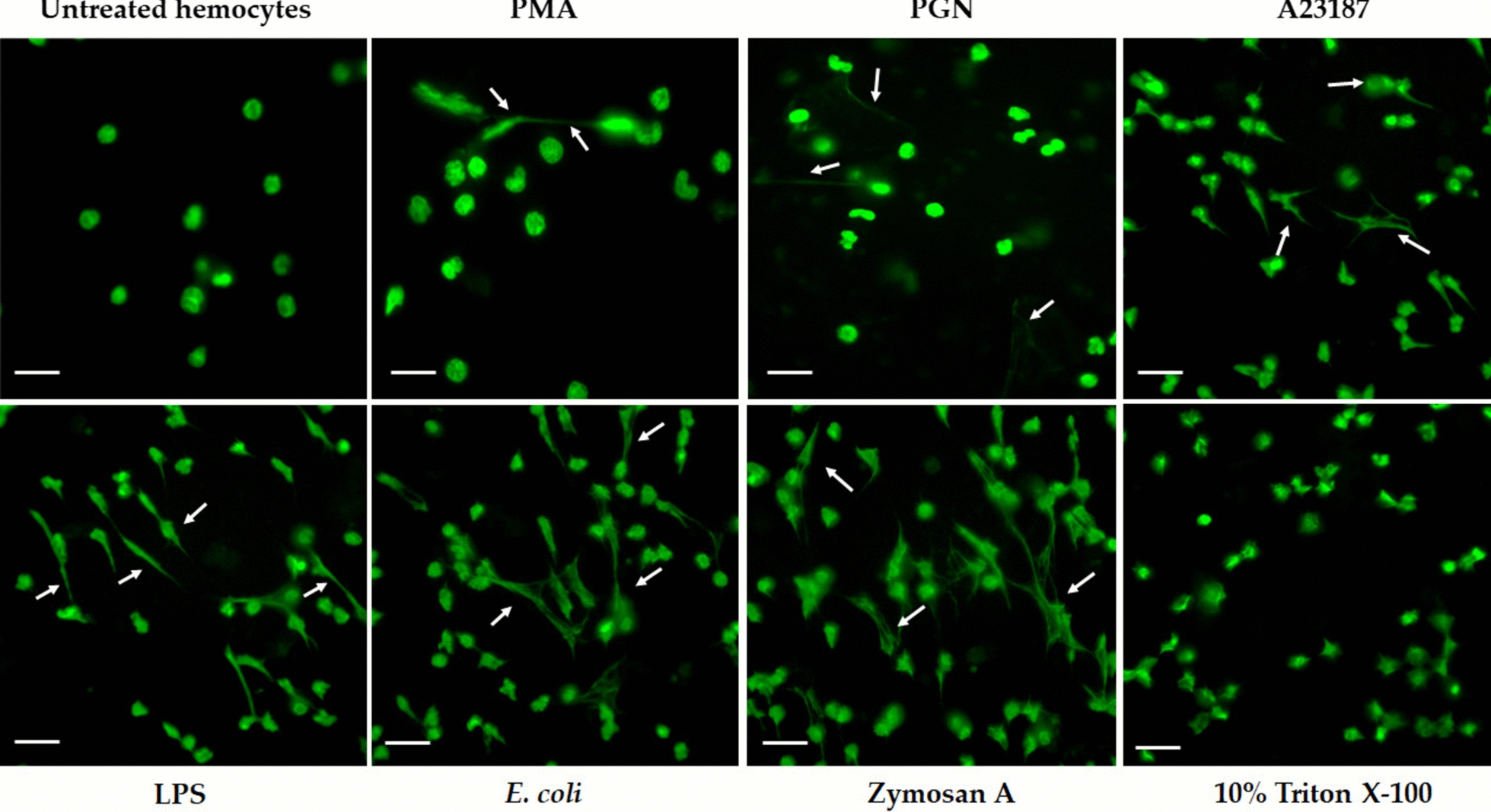
LCSM visualization of DNA fibers by 4′,6-diamidino-2-phenylindole (DAPI) staining. The tick hemocytes released DNA fibers (indicated by white arrows) after 30 min of incubation with all inducers, compared with untreated hemocytes. All micrographs are representative of independent experiments (*n* = 3) with three internal repetitions. Scale bar, 20 μm

The LSCM showed that hemocytes treated with different inducers released chromatin after 30 min of treatment. First, fluorescence in untreated hemocytes showed an intact and unsegmented nucleus, with a classical round morphology typical of quiescent cells. However, the tick hemocytes treated with different inducers appeared amorphous and distended, with DNA filamentous projections being released, and losing their nuclear integrity (Fig. 4). Particularly, the tick hemocytes treated with 0.050 µM PMA induced the release of long fine fibers formed by DNA, similar to classical ETs structures. In turn, the tick hemocytes incubated with 20 µg/mL PGN and 20 µM A23187 released short and thin DNA fibers. In contrast, the treatments with 0.01 µM zymosan A, 20 µg/mL LPS, and 7.5 × 10^5^* E. coli* cells showed a significant number of hemocytes releasing thin and long DNA fibers. Lastly, the hemocytes treated with 10% Triton X-100 did not release filamentous fibers but, instead, a diffuse release of chromatin around the cells was observed, which could indicate a disintegrated nucleus (Fig. 4).

**Fig. 5:**
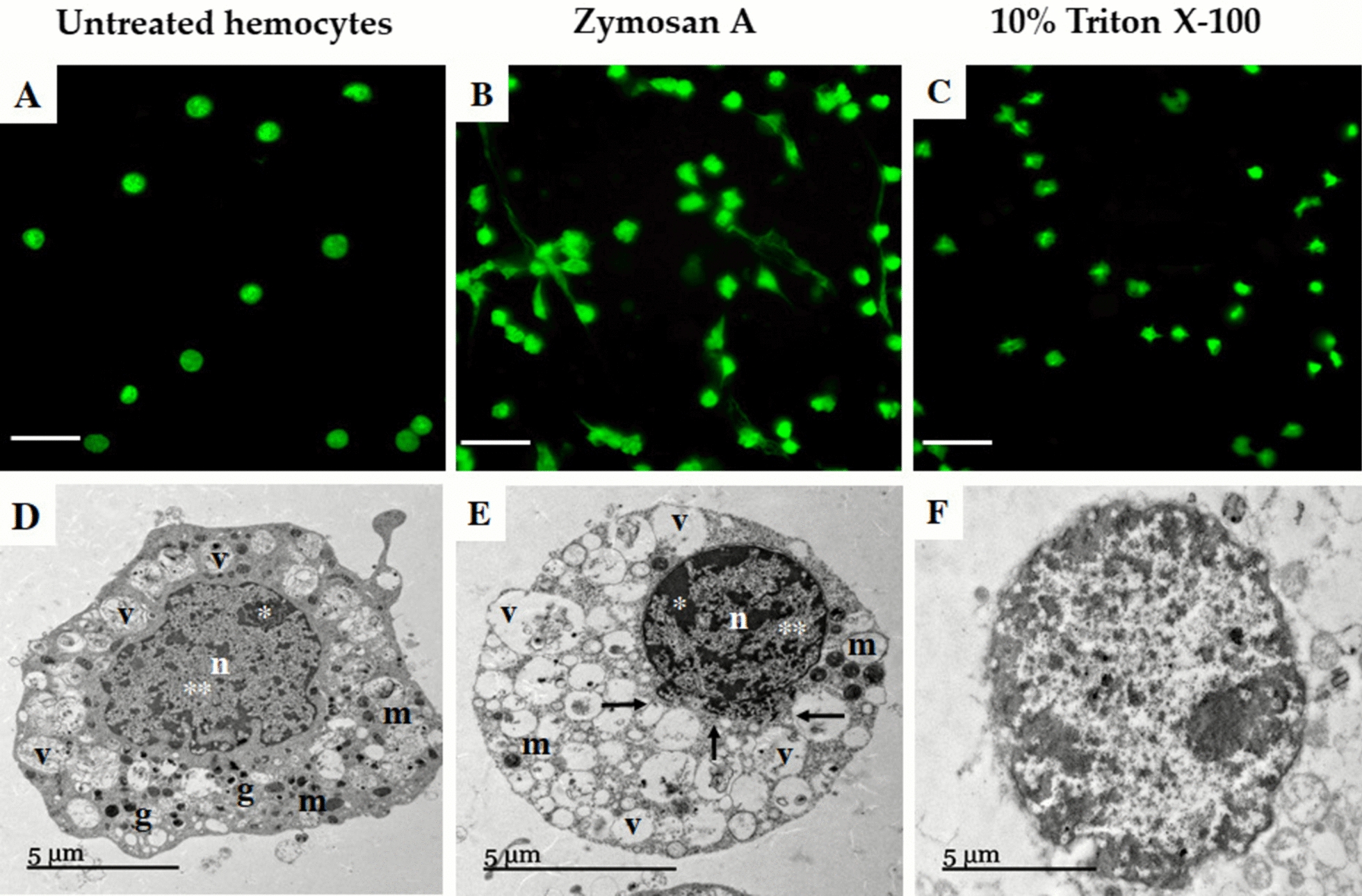
Representative LCSM and TEM micrographies of tick hemocytes treated with zymosan A at early times before chromatin release (15 min). LSCM hemocytes stained with DAPI: **A** untreated; **B** zymosan A (0.01 µM); and **C** 10% Triton X-100 (scale bar, 20 μm). TEM: **D** untreated; **E** zymosan A (0.01 µM); and **F** 10% Triton X-100; (n, nuclei; v, vacuoles; m, mitochondria; g, electron-dense granules; ^*^heterochromatin; ^**^euchromatin)

After visualizing the optimal chromatin release at 30 min, we decided to treat the hemocytes with the most effective inducer, aiming to observe cell morphology using LCSM and TEM before initiating the maximum chromatin release. LCSM analysis of hemocytes treated with 0.01 µM zymosan A at 15 min revealed long DNA fibers, as previously described. In addition, in untreated hemocytes, the chromatin is compact within a nuclear structure. Diffuse chromatin was observed in those hemocytes treated with 10% Triton X-100 (Fig. 5A, B, C). Regarding ultrastructural analysis by TEM, particular characteristics were revealed in hemocytes treated with 0.01 µM zymosan A at the early point of 15 min, in comparison to controls. The results showed that untreated hemocytes had an intact nucleus with a clear distinction between euchromatin and heterochromatin, as well as some membranous organelles (mitochondria), electron-dense granules, and vacuoles (Fig. 5D). Notably, in the ultrastructure of hemocytes treated with zymosan A, a lack of organelles, an increase in the number of cytoplasmic vacuoles, and material contained in the nucleus were observed. In addition, a partial nuclear envelope disruption, with a unidirectional release of chromatin, was observed (Fig. 5E). Finally, in those tick hemocytes treated with 10% Triton X-100, the diffuse chromatin was dispersed throughout the cytoplasm, organelles were not well defined, and there was an increase in vacuolization and dispersion of cellular content throughout the cytoplasm (Fig. 5F).

## Discussion

NETosis is a mechanism discovered in mammalian neutrophils; however, it has been demonstrated that other cellular types are also capable of producing extracellular traps (ETosis), including macrophages, PMNs, and even chicken heterophils and fish phagocytes [8, 9, 25–27]. Currently, the ETosis in invertebrates remains unclear; however, evidence has revealed the existence of this mechanism in the hemocytes of moths, shrimp, shore crabs, and bivalve mollusks, but there is no evidence in ticks to date [7, 10, 12, 28]. In addition, the ETosis has been hypothesized as a defense cell strategy against pathogens, which is evolutionarily conserved [9]. In this regard, ETosis could represent a key defense mechanism against diverse infections, considering that the tick life cycle involves a constant exposure to natural enemies, including the pathogens they transmit.

In this work, we assessed chromatin release in tick hemocytes in response to chemical and biological ETosis inducers, as well as bacterial cells. Our results showed that *R. microplus* hemocytes can release chromatin in response to severe stimuli, which is consistent with ETosis, supporting the existence of a defense immune mechanism conserved among arthropod species. Here, we used PMA and A23187 to assess chromatin release in tick hemocytes with classical NETosis inducers. Moreover, we found that chromatin release is time- and concentration-dependent for both inducers, suggesting that tick hemocytes can activate NOX-dependent or -independent ETosis, as has been reported for cnidarians, crustaceans, annelids, and even in bivalve hemocytes [9, 12, 29, 30]. Interestingly, in the oyster *Crassostrea gigas* and the mussel *Mytilus galloprovincialis*, reports indicate that PMA does not induce the release of ETs [11, 12]. This fact supports the idea that the ET formation by PMA depends on the dose, timing, and species under analysis [12].

However, LPS and PGN are the predominant stress-supporting structures of the cell envelope in bacteria and are also potent stimulators of the immunological response, including NETosis [8, 31, 32]. In addition, it has been reported that LPS from various bacterial sources induces a selective release of extracellular traps. Still, it is also known that different types of PGN are recognized by invertebrate hemocytes [33, 34]. In arthropods, LPS and PGN can activate humoral immune mechanisms, including amidase activity, ROS and NOS production, coagulation, melanization, and the induction of antimicrobial peptides via the Toll and Imd pathways [35]. However, the existence or activation of ETosis by LPS and PGN has never been reported in ticks. Notably, we found that LPS and PGN induced chromatin release in tick hemocytes in a concentration- and time-dependent manner, suggesting that this response could be modulated by the cell envelope composition (Gram-negative or Gram-positive bacteria). Remarkably, Pieterse et al. [33] found that LPS is capable of inducing ROS-dependent and suicidal NETosis in human neutrophils. Our results showed that LPS induces chromatin release; therefore, we do not rule out the possibility that this release is related to a similar ETosis pathway in tick hemocytes.

As mentioned before, ETosis reports and inducers in invertebrates are scarce; however, ETosis in shrimp hemocytes has been reported, where bacteria such as *E. coli* induced chromatin release [7, 36]. As is known, *R. microplus* has a microbiome with a wide range of bacterial diversity, and *E. coli* is not considered part of it; hence, it is interesting to us to utilize it as a source of microbe-associated molecular patterns (MAMPs) to induce ETosis in tick hemocytes [37–39]. In this regard, we observed that chromatin release is directly proportional to the number of *E. coli* cells used as inducers in a time-dependent manner. Consequently, we hypothesize that the immune response mediated by ETosis can distinguish between bacteria that form its microbiome, such as symbionts, commensals, and tick-borne pathogens (TBPs) (*Anaplasma marginale* or *Babesia bigemina* and *Babesia bovis)*, and those that could be pathogenic to the tick [40]. In addition, the *E. coli* ETosis induction observed in our results may be comparable to that reported in shrimp *Litopenaeus vannamei*, where *E. coli* is entrapped by entanglement in the DNA fibers, which exhibit an antibacterial effect [7].

However, fungi are considered the main natural enemy of arthropods, and they have even been proposed as an entomopathogenic control strategy [41]. Zymosan is a fungal substance composed of β-glucan and mannan, the typical components of fungal cell walls, involved in several innate immune responses, including ETosis activation. In addition, in arthropods, zymosan is recognized by pattern recognition receptors (PRRs), such as C-type lectins (mannose receptors) and Toll-like receptors (TLRs) [42, 43]. Our results showed that the chromatin release induced by zymosan A was higher than that of the other inducers and even comparable to the positive control of DNA released by cellular lysis (10% Triton X-100). According to Romero et al. [12], in bivalve mollusks such as *Mytilus galloprovincialis*, the treatment of hemocytes with zymosan A induces the release of ETs, and, mainly, this process is conducted by the NOX-dependent pathway, which results in NADPH-mediated ROS production that is necessary for ET activation. Similarly, in the bivalve *C. gigas*, the ETosis is also driven by zymosan A, leading to the activation of the NOX-dependent pathway [11]. Therefore, our results suggest that zymosan A may induce chromatin release in tick hemocytes by a pathway similar to that occurring in mollusks.

An interesting topic to analyze was gene expression of the *pxn* gene, considered an *mpo* homolog. MPO, a heme-containing peroxidase with a broad bactericidal ability, is key in NETosis activation [44]. In arthropods, PXN exhibits both peroxidase activity and adhesive cell properties with a significant role in the innate immune response [45–48]. Thus, we evaluate the *pxn* expression in tick hemocytes in response to various ETosis inducers. Our results showed a gene overexpression in all treatments, which is consistent with Shanthi et al. [49], who found a differential expression of *pxn* in *Fenneropenaeus indicus* hemocytes in response to treatment with the peptidoglycan of *Vibrio harveyi,* and with Dong et al. [50], who found patterns of *pxn* overexpression in response to challenge with different bacteria. It is worth highlighting that in invertebrates, *pxn* has been reported to respond to various microbial stimuli; however, its response to NETosis inducers, such as PMA and A23187, has not been evaluated. Interestingly, our results could be related to a gene expression pattern exclusive to PMA and A23187, as observed by Khan et al. [51], who reported that transcriptional firing occurs during NETosis, and more loci are transcribed in A23187-mediated NETosis compared with PMA-mediated NETosis. Thus, we hypothesize that *pxn* could be related to this transcriptional pattern and may play a role in the release of chromatin from tick hemocytes, considering that the release of extracellular traps is dependent on species, stimuli, and inducers [4].

Lastly, in general, the LCSM visualizations of tick hemocytes treated with inducers showed chromatin release, accompanied by the formation of fine fibers composed of DNA similar to the fibers released during NETosis. Notably, we observed differences in the length of the fibers, which varied according to the inducer. In addition, TEM visualizations revealed morphological and structural changes that may be part of the cell preparation before the traps’ release. These findings support the idea that chromatin released in tick hemocytes is a regulated mechanism rather than a consequence of cell lysis.

Although the characterization of phagocytic activity and humoral response has been reported in ticks, the immunological mechanism of ETosis has not been reported. To date, our work has demonstrated the release of chromatin from tick hemocytes in response to various stimuli. This finding demonstrates the probable formation of extracellular traps in tick hemocytes in a highly regulated manner and, consequently, reveals the existence of the ETosis mechanism in *R. microplus*. The data generated in this work expand the repertoire of innate immune responses known in these ectoparasites. Finally, this approach represents an important step toward understanding the tick’s immune response, and further research could focus on identifying genes and molecules that participate in ETosis activation and regulation in tick hemocytes, as well as exploring whether some pathogens, such as *Borrelia*, *Babesia*, or *Anaplasma* spp., can evade or inhibit this mechanism. With this contribution, new areas of research are opening up to understand the molecular mechanisms that govern this process, which we are currently working on. However, the findings of this work require more in-depth molecular and immunological approaches for a more precise characterization of the ETosis mechanism present in ticks.

## Conclusions 

The present study identified the chromatin release in tick hemocytes in response to chemical, biological, and bacterial inducers. Further study revealed the release of classical DNA fibers in tick hemocytes, as it has been observed in neutrophils undergoing NETosis. Our findings take us a step forward in the study of the tick’s immune response and set the basis for the exploration of the molecular mechanisms that regulate this process.

## Data Availability

The datasets used and/or analyzed during the current study are available from the corresponding author on reasonable request.

## Abbreviations

PMN: Polymorphonuclear
NETs: Neutrophil extracellular traps
ETs: Extracellular traps
ROS: Reactive oxygen species
NE: Neutrophil elastase
MPO: Myeloperoxidase
BPIF2: BPI fold containing family B, member 2
NOX-dependent: NADPH-oxidase-dependent
PMA: Phorbol myristate acetate
A23187: Calcium ionophore
PGN: Peptidoglycan
LPS: Lipopolysaccharide
UV: Ultraviolet
PXN: Peroxinectin
LCSM: Laser scanning confocal microscopy
TEM: Transmission electron microscopy
RFU: Relative fluorescence units
MAMPs: Microbe-associated molecular patterns
PRRs: Pattern recognition receptors
TLRs: Toll-like receptors
